# Germline Sequencing of Familial and Sporadic Early-Onset Colorectal Cancer: A Novel Pattern of Genes

**DOI:** 10.3390/ijms26104672

**Published:** 2025-05-14

**Authors:** Pierre Vande Perre, Ayman Al Saati, Bastien Cabarrou, Julien Plenecassagnes, Julia Gilhodes, Nils Monselet, Norbert Lignon, Thomas Filleron, Carine Villarzel, Laure Gourdain, Janick Selves, Mathilde Martinez, Edith Chipoulet, Gaëlle Collet, Ludovic Mallet, Delphine Bonnet, Rosine Guimbaud, Christine Toulas

**Affiliations:** 1 Oncogenetics Laboratory, Oncopole Claudius Regaud, IUCT-Oncopole, 31059 Toulouse, France; vandeperre.pierre@iuct-oncopole.fr (P.V.P.); alsaati.ayman@iuct-oncopole.fr (A.A.S.);; 2 DIAD Team, INSERM U1037, Centre de Recherches en Cancérologie de Toulouse, 31100 Toulouse, France; 3 Oncogenetics Department, Oncopole Claudius Regaud, IUCT-Oncopole, 31059 Toulouse, France; lignon.norbert@iuct-oncopole.fr (N.L.); chipoulet.edith@iuct-oncopole.fr (E.C.); bonnet.d@chu-toulouse.fr (D.B.); guimbaud.rosine@iuct-oncopole.fr (R.G.); 4 Faculté de Santé, Université de Toulouse, 31400 Toulouse, France; 5 Biostatistics & Health Data Science Unit, Oncopole Claudius Regaud, IUCT-Oncopole, 31059 Toulouse, France; cabarrou.bastien@iuct-oncopole.fr (B.C.); filleron.thomas@iuct-oncopole.fr (T.F.); 6 Bioinformatic Department, Oncopole Claudius Regaud, IUCT-Oncopole, 31059 Toulouse, France; plenecassagnes.julien@iuct-oncopole.fr (J.P.); mallet.ludovic@iuct-oncopole.fr (L.M.); 7 Pathology Laboratory, Oncopole Claudius Regaud, IUCT-Oncopole, 31059 Toulouse, France; selves.janick@iuct-oncopole.fr; 8 Oncology Department, Clinique Pasteur, 31076 Toulouse, France; mmartinez@clinique-pasteur.com; 9 CHU de Toulouse, 31059 Toulouse, France

**Keywords:** early-onset colorectal cancer, germline variants, NGS panel, molecular genetics of EOCRC

## Abstract

The majority of early-onset colorectal cancers (EOCRCs) are not substantiated by germline variants in the main CRC predisposition genes (the “DIGE” panel). To identify potentially novel EOCRC-specific predisposition genes, we analyzed 585 cancer pathway genes in an EOCRC patient cohort (n = 87, diagnosis ≤ 40 years, DIGE-), and compared their variant spectrum to the GnomAD cancer-free database. We identified high-impact variants (HVs) in 15 genes significantly over-represented in EOCRC. Among the 32 unrelated patients with a CRC family history (i.e., with a potentially dominant transmission pattern), 9 presented HVs in ten genes, four of which had a DNA repair function. We subsequently sequenced these 15 genes in a cohort of 82 late-onset CRCs (diagnosis ≥ 50 years, DIGE-) and found variants in 11 of these genes to be specific to EOCRC. We then screened these 11 genes in our patient database (n = 6482), which only contained 2% of EOCRCs (DIGE-), and identified two other EOCRC cases diagnosed after our cohort constitution, with HVs in *RECQL4* and *NUTM1*. Altogether, we found that 37.5% and 18.75% of patients carrying heterozygous *NUTM1* and *RECQL4* HVs, respectively, in our database were diagnosed with EOCRC. Our work has identified a pattern of germline variants not previously associated with EOCRC.

## 1. Introduction

Early-onset colorectal cancer (EOCRC) is a rare disease, characterized by late diagnosis/late-stage disease and left-sided primary tumors [1,2,3,4]. EOCRC is more prevalent in men and associated with a family history of colorectal cancer (CRC), inflammatory bowel disease, alcohol and tobacco use, a sedentary lifestyle, and obesity (body mass index ≥ 30) [5,6]. It is generally accepted that 5–10% of familial CRCs are caused by a genetic predisposition transmitted by a Mendelian pattern of inheritance, as Lynch syndrome and familial polyposis syndromes [7]. A French consensus colorectal gene panel (DIGE panel) [8] is therefore routinely used identifying germline pathogenic (PV) or likely pathogenic (LPV) variants or copy number variants (CNV, deletions or duplications) in 20% of EOCRC cases. Multi-gene testing [9,10,11,12], or whole exome sequencing (WES) [13,14,15,16,17,18,19], have been used to identify candidate genes for EOCRC risk. However, these studies have either tested small gene panels in large patient cohorts based on relatively non-selective inclusion criteria [9,10,11] or performed large molecular analyses (WES) on smaller cohorts (between 16 and 51 patients) [13,14,17,19].

The aim of the current study was therefore to identify additional EOCRC predisposition genes by sequencing 585 genes in a highly selective EOCRC cohort (age of diagnosis ≤ 40 years age) DIGE- (n = 87), with and without a CRC family history. We then compared this EOCRC gene variant’s profile to a late-onset CRC patient cohort DIGE- (LOCRC, diagnosed after 50 years of age, n = 82), and we screened the candidate genes in our local NGS patient database to search for variants in these new genes in additional EOCRC cases.

## 2. Results

### 2.1. EOCRC Cohort Characteristics

The DIGE-, EOCRC (n = 87) and LOCRC (n = 82) cohorts had a median age at diagnosis of 34 and 62.5 years, respectively (Table 1), a sex ratio (F/M) of 1.35 (*p* = 0.197 compared to LOCRC cohort, see Table 1). The EOCRC cohort characteristics were consistent with the literature in terms of distal disease (43.5% sigmoid or rectum involvement) or metastasis (23.5% metastatic disease) [9,10,11,13,15,18,19], and differed significantly from the LOCRC cohort (*p* < 0.002). As described in the literature in other EOCRC cohorts, our EOCRC cases were mainly microsatellite stable (MSS) or had low microsatellite instability (MSI-L) (76.2%) and were therefore mismatch repair proficient (pMMR), whereas LOCRCs had high microsatellite instability (MSI-H) and were mismatch repair deficient (dMMR).

Most patients had no additional medical history of cancer excluding EOCRC (87.4%) (Table 1).

Only 38.1% of all EOCRCs had a CRC family history, 34.4% of these with an affected first-degree and 75.0% a second-degree relative. This distribution differed significantly in the LOCRC cohort, with 75.8% (*p* < 0.001) and 39.4% (*p* = 0.004) having an affected first- and second-degree relative, respectively. This was consistent with previous studies reporting a first-degree family history in 15.0% (3/20) and 13.6% (3/22) of EOCRC cases [13,19].

### 2.2. Shortlisting of Germline High-Impact Variants in EOCRC Patients

**Figure 1:**
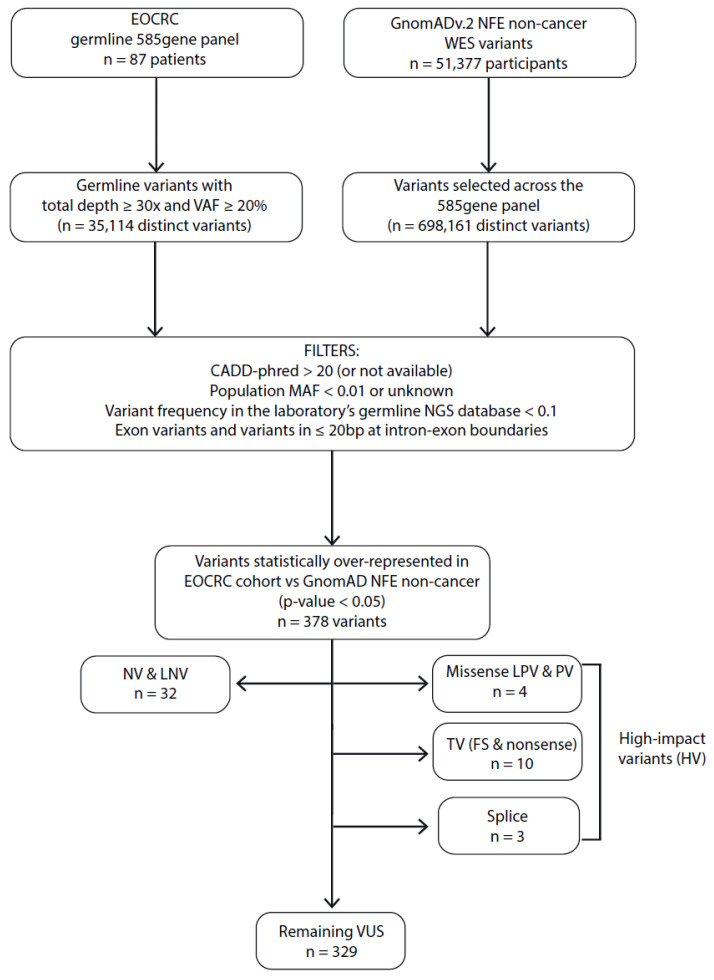
Filtering process to identify EOCRC susceptibility variants for the monogenic dominant hypothesis. MAF: minor allele frequency; VAF: variant allele frequency; bp: base pair; NFE: Non-Finnish European; WES: whole exome sequencing; EOCRC: early-onset colorectal cancer; PV: pathogenic variant; LPV: likely pathogenic variant; VUS: variant of unknown significance; LNV: likely neutral variant; NV: neutral variant; TV: truncating variants (frameshift (FS) and non-sense variants); CADD-phred: Combined Annotation Dependent Depletion score.

**Figure 2:**
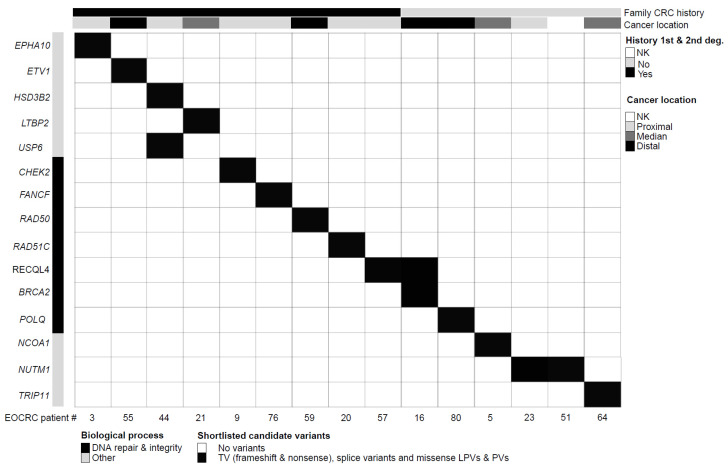
Clinical and molecular features of EOCRC patients with germline HVs. Each column corresponds to a patient (patient number specified). The upper section provides patient clinical features. Genes are grouped by biological processes (gene ontology). The upper section details patient clinical characteristics (1st- or 2nd-degree family history of colorectal cancer, anatomical digestive tract location of the disease). EOCRC: early-onset colorectal cancer; deg.: degree of kinship; NK: not known/not available; TV: truncating variants; PV: pathogenic variant; LPV: likely pathogenic variant.

By sequencing germline DNA for 87 EOCRC patients and filtering variants on a 585-gene panel (Figure 1 and Supporting Information Table S1), we identified 10 truncating variants (TVs i.e., frameshift and non-sense variant), 4 missense variants identified as LPVs or PVs in the ClinVar database, and 3 splice variants (SVs) in 15 different genes over-represented in our EOCRC population (adjusted *p*-value < 0.05) (Figure 2 and Table 2), while 329 variants were classified as of unknown significance (VUS). Among the 15 EOCRC patients with high-impact variants (HVs: TVs, SVs, and variants known to be PVs/LPVs), none carried VUS in DIGE panel genes, with the exception of EOCRC#16 with a VUS in the exonuclease domain of *POLD1* (Supporting Information Table S2).

Even though the relatively young age of the EOCRC cohort makes clonal hematopoiesis (CH) an unlikely rationale, we checked that the 15 EOCRC HVs were not included in the list of frequently mutated genes in CH [20], and that the variant allele frequencies (VAFs) were >35% (Figure 2 and Table 2). Patient EOCRC#09 carried a *CHEK2* gene variant included in the CH list, with a VAF of 47%, which seemed too high to be consistent with CH.

We pursued our analysis by successively investigating a dominant hypothesis for EOCRC, as well as recessive and oligogenic hypotheses.

### 2.3. Investigating a Dominant Transmission Pattern of EOCRC Predisposition

In the EOCRC cohort (n = 87), we identified 15 EOCRC patients carrying statistically over-represented HVs (*p* < 0.05). Nine of them, presenting a family history of CRC, carried HVs in 10 distinct genes (Figure 2 and Table 2), including genes involved in DNA repair pathways (*CHEK2*, *FANCF*, *RAD50*, and *RAD51C*) and DNA integrity (*RECQL4*). Among these patients, EOCRC#09 (*CHEK2* variant) had a maternal family history of breast and pancreatic cancer (2nd- and 3rd-degree relatives). His maternal grandmother was diagnosed with both CRC and breast cancer at 65 years of age. *CHEK2* germline PVs/LPVs are considered as moderate-risk factors for breast cancer. Patient EOCRC#57 (*RECQL4* variant) presented with a mucinous adenocarcinoma of the ascending colon at the age of 32 and her father was diagnosed with CRC at the age of 64. EOCRC#57 was heterozygous for the *RECQL4* PV (no evidence of compound heterozygosity) and did not exhibit any syndrome features of *RECQL4*-associated recessive syndromes as Rothmund–Thomson (RTS, MIM #268400), RAPADILINO (MIM #266280) and Baller–Gerold (BGS, MIM #218600)) [21]. The remaining HVs in the EOCRCs with a family CRC history mapped to genes involved in steroid biosynthesis (*HSD3B2*), transcription regulation (*ETV1*), and other cellular pathways (*EPHA10*, *LTBP2*, and *USP6*). Patient EOCRC#44 who carries HVs in both *HSB3B2* and *USP6* genes presented with adenocarcinoma of the ascending colon at the age of 36 and his father was reported with CRC (unknown age at diagnosis).

The remaining six EOCRC patients with HVs had no family history of CRC. They carried seven variants in six distinct genes (Figure 2 and Table 2), including DNA repair (*BRCA2* and *POLQ*) and DNA integrity genes (*RECLQL4*). Patient EOCRC#16 with both *BRCA2* (a PV inherited from her father) and *RECQL4* variants presented with a rectal adenocarcinoma at 39 years of age. The remaining variants mapped to genes involved in transcription regulation (*NCOA1*), Golgi apparatus trafficking (*TRIP11*), and possibly cell proliferation by modulation of TERT expression (*NUTM1*). In addition to the EOCRC initially reported at the age of 37, patient EOCRC#64 (*TRIP11* variant) was diagnosed with pineal dysgerminoma at the age of 21 and relapsed with a malignant germinoma. Patients EOCRC#23 (adenocarcinoma of the appendix at 36 years of age) and EOCRC#51 (colon cancer diagnosed at 38), from unrelated families, carried the same TV in *NUTM1*.

To investigate whether variants in these genes are associated with early onset, we screened these 15 genes in the LOCRC cohort (Table 3 and Supporting Information Table S3). Four HVs (in the *CHEK2*, *FANCF*, *POLQ*, and *TRIP11* genes), and 28 VUSs were identified in these 15 genes in LOCRC. Eleven genes were completely devoid of HVs in the LOCRC cohort. Six LOCRC patients (7.3%) carried HVs in these genes, compared to 15 EOCRC patients (17.2%). Patients LOCRC#69 and LOCRC#74 carried the same TV in *CHEK2*, but only LOCRC#69 had a CRC family history. Whilst two EOCRC patients carried the same HV in the *RECQL4* gene (one with and the other without a CRC family history) and two other EOCRC patients (both with a CRC family history) presented with the same HV in the *NUTM1* genes, none of the LOCRC patients had HVs in either of these genes. These analyses highlight HV profile differences between EOCRC and LOCRC patients.

To further explore whether variants of these 11 genes might be associated with EOCRC, we screened our germline NGS database for HVs in 10 of these genes (*BRCA2* was excluded) (Table 4). Approximately 80% of the patients included in our local NGS database (n = 6482) were tested for breast/ovarian cancer, 10–15% for polyposis or CRC (of which 2% are DIGE- EOCRC patients (n = 130)), and 5–10% for other cancers or rare cancer-free members of high-risk families. Of the 130 EOCRC DIGE- patients in this database, 87 were included in our current EOCRC cohort and 43 had been integrated in this database between the interval between the end of cohort inclusion and the screening of the NGS database. Indeed, *BRCA2* PVs are not associated with CRC [22], and the frequency of *BRCA2* HVs in the EOCRC cohort does not appear to be higher than in the general population. HVs in *NUTM1*, *RECQL4*, *RAD50*, *RAD51C*, *EPHA10*, *ETV1*, *LTBP2*, *NCOA1*, and *USP6* genes were identified in 103 “new” database patients (Table 4) but not to *HSD3B2*. No additional EOCRC case was identified among the screened database individuals with HVs in *RAD50*, *RAD51C*, *EPHA10*, *ETV1*, *LTBP2*, *NCOA1*, and *USP6*). In contrast, additional EOCRC cases were identified by screening the *NUTM1* and *RECQL4* genes. A more recent EOCRC patient (DIGE-) was identified with *RECQL4* c.2547_2548del, p.(Phe850Profs*33) TV, and is subsequently referred to as “EOCRC#88new”. She had a rectal cancer at the age of 39 but had no family history of CRC. The *NUTM1* TV c.3406C>T, p.(Arg1136*) was also identified in an additional EOCRC (DIGE-) patient (“EOCRC#89new”). This patient presented with adenocarcinoma of the descending colon at the age of 31. Her mother and her only maternal aunt, respectively, developed CRC at 51 and 52 years of age. Finally, 37.5% (3/8) of heterozygous *NUTM1* TV carriers and 18.75% (3/16) of heterozygous *RECQL4* TV or SV carriers in our database have been diagnosed with EOCRC, suggesting the involvement of these genes in EOCRC risk.

### 2.4. Investigating a Monogenic Recessive Transmission and an Oligogenic Pattern of EOCRC Predisposition

**Figure 3:**
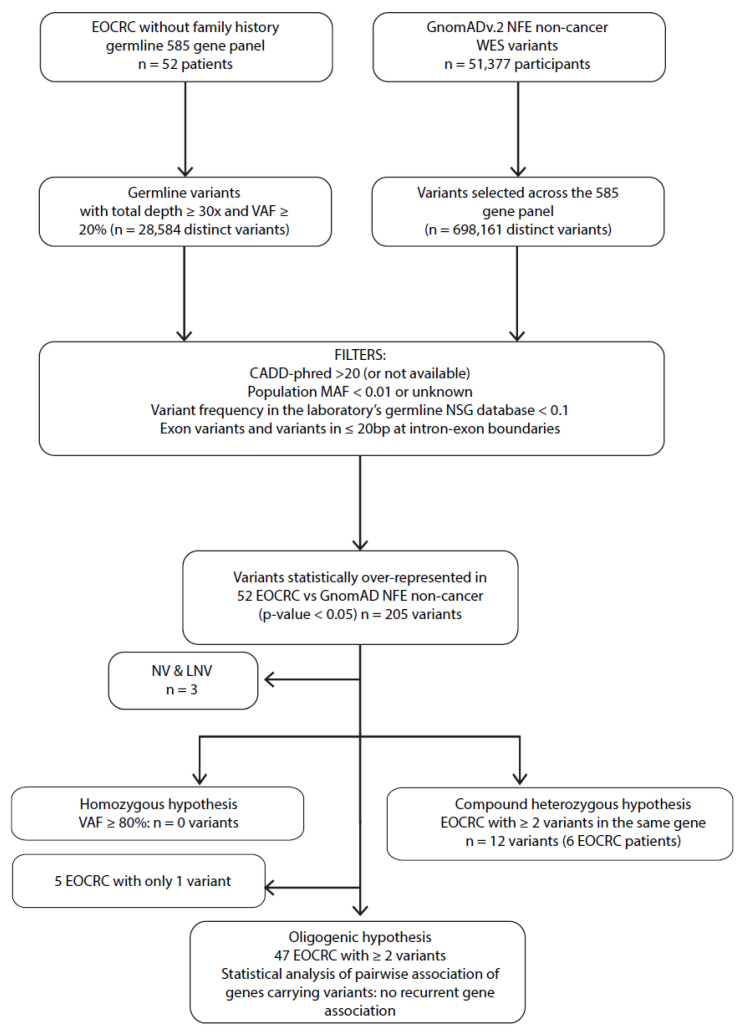
Filtering process to identify EOCRC susceptibility variants for monogenic recessives (homozygous and compound heterozygous) and oligogenic approaches. MAF: minor allele frequency; VAF: variant allele frequency; bp: base pair; NFE: non-Finnish European; WES: whole exome sequencing; EOCRC: early-onset colorectal cancer; LNV: likely neutral variant; NV: neutral variant; CADD-phred: Combined Annotation Dependent Depletion score.

In the subgroup of EOCRC patients without a CRC family history (n = 52) (Figure 3), no significant homozygous variants (VAF ≥ 80%) were identified compared to the GnomAD of the subgroup non-cancer of non-Finnish European origin (NFE). To investigate the recessive compound heterozygous hypothesis, we reported five EOCRC patients carrying at least two variants of the same gene (*CNTRL* (patient EOCRC#5 and EOCRC#72), *POLD1* (patient EOCRC#18), *RECQL4* (patient EOCRC#16), and *TCF12* (patient EOCRC#12) (Figure 3, Supporting Information Table S2). These four genes were then screened in the LOCRC cohort and the whole NGS database without identified patients carrying a recurrent pattern of multiple or homozygous variants in these genes (Supporting Information Table S4). Finally, we wanted to investigate a possible recurrent pattern of gene association among sporadic patients (oligogenic hypothesis). However, the limited size of our population and the major limitations of large but targeted sequencing did not allow us to conclude for an oligogenic transmission pattern of EOCRC predisposition (Supporting Information Table S5).

## 3. Discussion

To identify potentially novel gene variants that predispose to EOCRC, we successively examined dominant, recessive, and oligogenic hypotheses as appropriate in the part of our cohort with (n = 32) and without (n = 52) a family history of CRC, analyzed on a 585-gene panel.

We identified HVs that were significantly over-represented in our EOCRC cohort compared to the GnomAD non-cancer NFE population, in the following 15 genes: *BRCA2*, *POLQ*, *RAD50*, *RAD51C*, *RECQL4*, *CHEK2*, *FANCF*, *EPHA10*, *ETV1*, *HSD3B2*, *LTBP2*, *NCOA1*, *NUTM1*, *TRIP11*, and *USP6*. This approach of statistical comparison between our cohort germline data and that of GnomAD NFE non-cancer substantially reduces the possibility of incidental findings. Previous studies investigating EOCRC populations at age cut-offs ranging from 40 to 55 years, either with small gene panels on larger populations [9,10,11], or by exome analysis on smaller populations [13,14,15,16,17,19], identified variants predominantly in DNA repair genes [9,10,11,12,16] and in putative novel cancer genes such as *EIF2AK4*, *PTPN12*, and *LRP6* [14,15]. Our findings are consistent with these studies since 50% of our candidate (HV) genes are involved in DNA repair pathways. Although *BRCA1* and *BRCA2* PVs/LPVs have previously been reported in 0.3 to 5% of EOCRC patients [9,10,11,16,18], the frequency of BRCA variants in EOCRC cohorts may simply reflect the frequency of these PVs/LPVs in the general population. A recent meta-analysis confirmed this conclusion by showing that carriers of *BRCA1/2* mutations are not at increased risk of CRC [22].

It is worth noting that most of the variants (five out of seven) identified in DNA repair genes occurred in EOCRC patients with a 1st- or 2nd-degree family history of CRC. DNA repair is the main pathway involved in various germline family cancer predispositions (as family breast cancer and colorectal cancer). However, among the subgroup of 32 EOCRC patients with a CRC family history, 4 additional patients carried variants in genes regulating TGF-β signaling (*LTBP2*), transcription (*ETV1*), cell–cell communication (*EPHA10*), steroid biosynthesis (*HSD3B2*), and vesicle-mediated transport (*USP6*), suggesting that cellular pathways other than DNA repair may also be involved in EOCRC risk.

It should be noted that no family analysis has been carried out for the HVs detected in this work, so the inherited or de novo status of these variants is unknown.

In the 52 EOCRC patients without a CRC family history, the recessive hypothesis was explored without identifying a homozygous variant of interest. Similarly, the search for compound heterozygosity was inconclusive and the oligogenic hypothesis could not be confirmed. In this EOCRC population without a family history of CRC, we also examined a dominant hypothesis by identifying six patients presented with HVs in six distinct genes, three patients with HVs in DNA repair genes (*BRCA2*, *POLQ*, and *RECQL4*), and four patients with variants in genes regulating other cellular functions such as transcription (*NCOA1*), Golgi trafficking (*TRIP11*), and TERT regulation (*NUTM1*). *POLQ* variants, a DNA polymerase with helicase activity involved in DNA repair, have been reported in early-onset breast cancer women [23], and seem to be associated (but not confirmed) to CRC risk [24]. To our knowledge, *TRIP11,* which maintains Golgi apparatus structure and interacts with thyroid hormone receptor beta, has not been involved in CRC oncogenesis. In contrast, NCOA1 (or SRC-1/RIP160) is one of the main transcription co-activators of nuclear receptors [25] and binds to β-catenin, a major actor of the Wnt pathway, extensively described in CRC oncogenesis [26].

We then attempted to determine whether this variant pattern is specific to the early onset of disease by analyzing the variant profile of these 15 genes of interest in a cohort composed of 82 LOCRC patients. Eleven out of the fifteen genes (*BRCA2*, *RAD50*, *RAD51C*, *RECQL4*, *EPHA10*, *ETV1*, *HSD3B2*, *LTBP2*, *NCOA1*, *NUTM1*, and *USP6*) carried HVs exclusively in the EOCRC cohort, suggesting a distinct germline background between EOCRC and LOCRC.

We next assessed whether the analysis of these 10 genes of interest (*BRCA2* excluded) could lead to the identification of other EOCRC patients in our local NGS database. No other EOCRC patient was detected among individuals with HVs in *RAD50*, *RAD51C*, and *USP6* genes. However, two other EOCRC cases diagnosed after the recruiting period of our cohort carried truncated variants in *NUTM1* and *RECQL4*. Both variants (three patients) of *NUTM1* identified in EOCRC patients in the current study are located in exon 8, and both variants (three patients) of *RECQL4* identified are located in the sequence encoding the helicase domain of the protein (exons 9 and 15). *RECQL4* (RecQ-Like Helicase 4) encodes a DNA helicase that controls recombination, replication, and DNA repair [27]. Bi-allelic *RECQL4* defects are implicated in autosomal recessive inheritance syndromes (RTS, RAPALIDINO, and BGS), but none of our *RECQL4* TVs heterozygous carriers showed clinical features of these conditions. Both *RECQL4* variants are located in the helicase domain of the protein [21,28,29,30,31]. A retrospective study of heterozygous *RECQL4* carriers from RTS families found no significant difference in cancer risk compared to the general population, but the locations of the *RECQL4* variants were not well defined in this study [32]. The *NUTM1* gene encodes the NUT protein (nuclear protein in testis, or C15orf55), which may up-regulate telomerase reverse transcriptase (TERT) expression by binding to the TERT promoter SP1 binding site [33]. Patients EOCRC#51 and EOCRC#23 carry the same heterozygous *NUTM1* variant predicted to truncating the NUT protein C-terminus involving the nuclear localization sequence [34]. An additional EOCRC#89 new patient carried a heterozygous variant of *NUTM1* also truncating the protein. Further studies are required to investigate the potential role of this C-terminal truncated NUTM1 protein in EOCRC oncogenesis. Interestingly, a previous genome-wide association study in a Korean population identified three loci associated with CRC; one of these included *NUTM1*, specifically the 20 amino acids before the C-terminus of NUTM1 (rs2279685, chr5:34649631) [35].

Heterozygous TVs in *NUTM1* and *RECQL4* appear to be significantly over-represented, in our NGS database, among individuals with a history of EOCRC, with 38.5% (3/8) of *NUTM1* and 18.75% (3/16) of *RECQL4* TV carriers diagnosed with EOCRC while the proportion of EOCRC patients (germline DIGE-) in our NGS database was 2% (130/6482). The EOCRC phenotype appears to be highly over-represented among the heterozygous *NUTM1* and *RECQL4* TV carriers.

To conclude, our study identifies distinct germline patterns of variants in 10 genes depending on the EOCRC patient family history with (*RAD50*, *RAD51C*, *RECQL4*, *EPHA10*, *ETV1*, *LTPB2*, *USP6* genes) or without (*NCOA1*, *NUTM1*, *TRIP11* genes) family history of CRC, absent in a population with a later CRC diagnosis (LOCRC). In addition, two genes of this pattern, *NUTM1* and *RECQL4*, led to identify other cases of EOCRC from our whole NGS database. This work paves the way for further studies (for example, functional testing) to determine the role of at least *NUTM1* and *RECQL4* genes in EOCRC risk and oncogenesis. These data, once confirmed by larger studies, would have potential application for EOCRC risk assessment and follow-up and for further treatments or preventions (as the use of low dose of aspirin to reduce CRC incidence).

## 4. Materials and Methods

### 4.1. Cohorts

All CRC (or small bowel cancer) patients referred to our institute between 2016 and 2021, DIGE-, were included in the analysis. We identified a DIGE- EOCRC cohort consisting of unrelated, adult patients (n = 87) initially diagnosed with either early-onset colorectal or small bowel cancer (≤40 years of age at diagnosis), and a DIGE- cohort of late-onset CRC (or small bowel cancer) patients diagnosed after 50 years of age (LOCRC, n = 82). All patients gave their written informed consent for the germline genetic analysis of cancer genes for both diagnostic and research purposes.

Small bowel and appendix cancer were accounted as proximal disease location due to their histology of digestive track adenocarcinoma: we have endeavored to remain exhaustive. These cases of small bowel and appendicular adenocarcinoma represent only a limited fraction of the studied cohorts (4.7% and 3.5% in EOCRC and 2.6% and 0% in LOCRC, respectively).

Tumor MMR and microsatellite status were determined from formalin-fixed colorectal or small bowel tumor tissue as previously described [36].

The clinicopathological characteristics of patients and their families were collected through the medical files from the genetic consultation.

### 4.2. Germline 585-Gene Panel Sequencing

Blood DNA was sequenced with the customized Comprehensive Cancer Panel (Roche, Bâle, Switzerland) of 585 cancer predisposition/cancer pathway genes (Supporting Information Table S6), and analyzed as described previously ([37] and Supporting Information Table S7). Variants with a total sequencing depth of ≥30x and a variant allele frequency (VAF) ≥20% were selected for further analyses.

### 4.3. Variant Analysis and Classification

All coding (SNVs, small insertions and deletions) and intron variants (located in ≤20 bp intron DNA at intron/exon boundaries) were further evaluated. Rare variants were filtered by their predicted pathogenicity score (Combined Annotation Dependent Depletion—CADD-Phred score [38] > 20 or not available), by their minor allele frequency (MAF) in GnomAD (whole base < 1%) and were identified in fewer than 10% of NGS database samples (Figure 1 and Figure 2). Copy number variants (CNVs), not available in GnomADv2, were not evaluated.

Variants significantly over-represented in the DIGE-EOCRC group were classified according to the ACMG (American College of Medical Genetics and Genomics) recommendations [39]. Neutral and likely neutral (NV/LNV) variants were excluded from the analysis. Statistically significant variants were confirmed by reviewing the BAM files with the Integrative Genomics Viewer (IGV). Potential splice variants (SVs) were considered if located in the exon or in the 2 base pairs immediately adjacent to the intron/exon boundary (i.e., −2, −1 or +1, +2 relative to the splice junction) and if splice effect impact was predicted by both SpliceAI and SPIP pipelines [40,41].

### 4.4. Control Population for Statistical Analysis of NGS Data

The NGS data from the EOCRC cohort were compared to the germline data from the GnomADv2 “non-cancer” subpopulation of NFE origin (51,377 patients) [42], restricted to the 585-gene target regions (F/M ratio close to 0.5; 85% of this control population being over 40 years of age).

### 4.5. Statistical Analysis

Categorical variables are expressed as frequencies and percentages and continuous variables as medians with ranges. Comparisons of the frequency of each variant between the DIGE- EOCRC and the GnomAD non-cancer NFE population were performed using the Fisher’s exact test with a Benjamini–Hochberg procedure for multiple testing. Comparisons between the DIGE- EOCRC and LOCRC groups were assessed using the Chi-squared or Fisher’s exact test for categorical variables and the Kruskal–Wallis test for continuous variables. All statistical tests were two-tailed and *p*-values < 0.05 were considered statistically significant. Statistical analyses were performed using R (v4.1.2) and the STATA software (v18) (Stata Corporation, College Station, TX, USA). Gene ontology term scoring and pathway enrichment analysis were performed with the g: Profiler [43].

### 4.6. Screening the Local NGS Database for Candidate Gene Variants

Our NGS database (n = 6482 patients) was screened for the presence of candidate HVs identified in the EOCRC cohort.

## Figures and Tables

**Table 1:** EOCRC (n = 87) and LOCRC (n = 82) patient characteristics.

	EOCRC (n = 87)	LOCRC (n = 82)	*p*-Value
Median age at cancer diagnosis (years) [range]	34 [20; 40]	62.5 [50; 85]	**<0.001**
Sex: women/men	50 (57.5%)/37 (42.5%)	39 (47.6%)/43 (52.4%)	0.197
Histology: adenocarcinoma			
Lieberkuhnian	46 (73.0%)	39 (69.6%)	
Colloid or mucinous	10 (15.9%)	9 (16.1%)	
Signet ring cell carcinoma	2 (3.2%)	3 (5.4%)	
Medullary	0	0	
Mixed Lieberkuhnian and colloid	3 (4.8%)	4 (7.1%)	
Mixed colloid and signet ring cell	1 (1.6%)	1 (1.8%)	
Mixed medullary and signet ring cell	1 (1.6%)	0	
Missing data	24	26	
Cancer by location			
Small bowel	4 (4.7%)	2 (2.6%)	
Appendix	3 (3.5%)	0	
Ascending colon	18 (21.2%)	39 (51.3%)	
Transverse colon	7 (8.2%)	1 (1.3%)	
Descending colon	16 (18.8%)	18 (23.7%)	
Sigmoid	19 (22.4%)	6 (7.9%)	
Rectum	18 (21.2%)	10 (13.2%)	
Missing data	2	6	
Location subclasses			**0.002**
“Proximal” (small bowel, appendix, ascending colon)	25 (29.4%)	41 (53.9%)	
“Medial” (transverse and descending colon)	23 (27.1%)	19 (25.0%)	
“Distal” (sigmoid, rectum)	37 (43.5%)	16 (21.1%)	
Colon without documented location	2	6	
Metastatic CRC at primary diagnosis			**0.002**
Yes	20 (23.5%)	4 (5.5%)	
No	65 (76.5%)	69 (94.5%)	
Missing data	2	9	
Tumor microsatellite instability			**<0.001**
MSS or MSI-L	48 (76.2%)	13 (19.4%)	
MSI-H	15 (23.8%)	54 (80.6%)	
Missing data	24	15	
Immunohistochemistry MMR protein expression			**<0.001**
pMMR	55 (79.7%)	14 (19.7%)	
dMMR	14 (20.3%)	57 (80.3%)	
Missing (or incomplete) IHC	18	11	
Methylation of the *MLH1* promoter			0.283
Yes	2 (22.2%)	20 (44.4%)	
No	7 (77.8%)	25 (55.6%)	
Missing data	78	37	
BRAF V600E mutation			0.160
Yes	3 (15.0%)	20 (44.4%)	
No	17 (85.0%)	25 (55.6%)	
Missing data	67	37	
CRC patients with an additional independent cancer history			**0.032**
Yes	11 (12.6%)	21 (25.6%)	
No	76 (87.4%)	61 (74.4%)	
Type(s) of independent cancer(s):			
Colorectal	2 (18.2%)	3 (14.3%)	
Small bowel NET	1 (9.1%)	0	
Cholangiocarcinoma	1 (9.1%)	0	
Prostate	1 (9.1%)	3 (14.3%)	
Lung	1 (9.1%)	1 (4.8%)	
Melanoma	2 (18.2%)	1 (4.8%)	
Breast	2 (18.2%)	4 (19.0%)	
Hematological malignancies (ALL, CLL)	1 (9.1%)	1 (4.8%)	
Hodgkin’s lymphoma	1 (9.1%)	0	
Skin lymphoma	0	1 (4.8%)	
Pineal dysgerminoma	1 (9.1%)	0	
Kidney	0	3 (14.3%)	
Pancreas	0	1 (4.8%)	
Bladder	0	1 (4.8%)	
Ovary	0	3 (14.3%)	
Adrenal cortex	0	1 (4.8%)	
Non-melanoma skin tumor	0	2 (9.5%)	
Endometrium	0	1 (4.8%)	
Thyroid	0	2 (9.5%)	
Family history of CRC			0.680
Yes	32 (38.1%)	33 (41.3%)	
No	52 (61.9%)	47 (58.8%)	
Missing data	3	2	
If yes:			
1st-degree relative	11 (34.4%)	25 (75.8%)	**<0.001**
2nd-degree relative	24 (75.0%)	13 (39.4%)	**0.004**

The number of patients (n), percentages (%, excluding any missing data), and medians [ranges] are listed for each category with *p*-values < 0.05 indicated in bold. EOCRC: early-onset colorectal cancer; IHC: immunohistochemistry; LOCRC: late-onset colorectal cancer; CRC: colorectal cancer; MMR: mismatch repair; dMMR: deficient MMR (if expression of one of the four MMR proteins is lost); pMMR: proficient MMR (tumors expressing the four MMR proteins); MSS: microsatellite stable; MSI: microsatellite instability (i.e., MSI-H (high) if two or more markers are affected, MSI-L (low) if only one of the five markers is concerned); ALL: acute lymphocytic leukemia; CLL: chronic lymphocytic leukemia; NET: neuroendocrine tumor.

**Table 2:** Molecular characteristics of germline HVs in EOCRC patients. *p*-values are specified for each variant.

Function	Gene	Patient ID	HGVS Nomenclature	VAF (%)	Adjusted *p*-Value	Medical CRC History (Age at Diagnosis)	
						Patient	Family
DNA repair	*BRCA2*	EOCRC#16	c.3847_3848del, p.(Val1283Lysfs*2)	53	0.0219	Rectum ADK (39)	No
	*CHEK2*	EOCRC#09	c.190G>A, p.(Glu64Lys)	47	0.0466	Ascending colon ADK (33)	Maternal GM: CRC (65) and paternal GF: CRC (67)
	*FANCF*	EOCRC#76	c.604del, p.(Leu202*)	54	0.0102	Ascending colon ADK (39)	Brother: CRC (57)
	*POLQ*	EOCRC#80	c.7537C>T, p.(Gln2513*)	49	0.0398	Sigmoid ADK (38)	No
	*RAD50*	EOCRC#59	c.130-1G>T	51	0.0093	Sigmoid ADK (34)	Father: CRC (72)
	*RAD51C*	EOCRC#20	c.773G>A, p.(Arg258His)	54	0.0134	ADK of the small bowel (28)	Paternal GM: CRC (73)
DNA helicase	*RECQL4*	EOCRC#57	c.1573del, p.(Cys525Alafs*33)	47	0.0093	Ascending colon ADK (32)	Father: CRC (64)
		EOCRC#16		36		Rectum ADK (39)	No
Transcription factor	*ETV1*	EOCRC#55	c.1378dup, p.(Met460Asnfs*80)	46	0.0144	Rectum ADK (39)	Father: RC (NA)
	*NCOA1*	EOCRC#05	c.4282dup, p.(Gln1428Profs*14)	51	0.0093	Ascending colon ADK (33)	No
Enzyme (steroid biosynthesis)	*HSD3B2*	EOCRC#44	c.776C>T, p.(Thr259Met)	45	0.0093	Ascending colon ADK (36)	Father: CRC (NA)
Microtubule/Golgi binding protein	*TRIP11*	EOCRC#64	c.5719+2T>C	49	0.0297	Descending colon ADK (37)	No
Transferase and tyrosine kinase activity	*EPHA10*	EOCRC#03	c.2446G>T, p.(Glu816*)	48	0.0146	Rectum ADK (36)	Maternal GM: CRC (56)
Deubiquitination and vesicle-mediated transport regulation	*USP6*	EOCRC#44	c.3228+1G>A	41	0.0394	Ascending colon ADK (36)	Father: CRC (NA)
TGFβ binding	*LTBP2*	EOCRC#21	c.4933C>T, p.(Arg1645Trp)	46	0.0202	Descending colon ADK (32)	2 Paternal uncles: CRC (75)
Regulator of the TERT pathway	*NUTM1*	EOCRC#51	c.2076_2077del, p.(Gly694Serfs*26)	46	0.0093	Appendix ADK (36)	No
		EOCRC#23		38		Colon cancer of unknown anatomical location (34)	No

ADK: adenocarcinoma; CRC: colorectal cancer; EOCRC: early-onset colorectal cancer (≤40 years of age at diagnosis); GM: grandmother; GF: grandfather; HV: high-impact variant i.e., truncating, splice or variants already classified as pathogenic/likely pathogenic variants in ClinVar database; LOCRC: late-onset colorectal cancer (≥50 years at diagnosis); NA: not available; RC: rectal cancer; VAF: variant allele frequency.

**Table 3:** Screening for HVs in 15 genes of interest in the LOCRC cohort revealed HVs in *CHEK2*, *FANCF*, *POLQ*, and *TRIP11* genes (no HV in *BRCA2*, *EPHA10*, *ETV1*, *HSD3B2*, *LTBP2*, *NCOA1*, *NUTM1*, *RAD50*, *RAD51C*, *RECQL4*, and *USP6*). *p*-values are specified for each variant.

Gene	Patient ID	HGVS Nomenclature	VAF (%)	Medical CRC History (Age at Diagnosis)	
				Patient	Family
*CHEK2*	LOCRC#69	c.591del, p.(Val198Phefs*7)	46	Colon cancer of unknown anatomical location (60)	Sister: CRC (46)
	LOCRC#74		49	ADK of the ascending colon (60 and 71)	No
*FANCF*	LOCRC#23	c.1087C>T, p.(Gln363*)	38	ADK of the descending colon (59)	No
*POLQ*	LOCRC#62	c.4262_4268del, p.(Ile1421Argfs*8)	45	ADK of the ascending colon (53)	No
	LOCRC#51		48	ADK of the ascending colon (60, 72 and 83)	Father: CRC (NA)
*TRIP11*	LOCRC#76	c.2467_2470del, p.(Arg823Valfs*15)	42	ADK of the ascending colon (55)	No

HV: high-impact variant, i.e., truncating, splice or variants already classified as pathogenic/likely pathogenic variants in ClinVar database; LOCRC: late-onset colorectal cancer (≥50 years at diagnosis); VAF: variant allele frequency; CRC: colorectal cancer; ADK: adenocarcinoma; NA: not available.

**Table 4:** Screening of our local NGS database for HVs in 10 candidate genes (n = 6482 patients, of which 130 are DIGE- EOCRCs patients).

Gene NM	HGVS Nomenclature	Cancer Personal History of Carrier (Number of Carriers)	
		CRC	Non-CRC Cancer
*NUTM1* *NM_001284292.1*	c.303_304del, p.(Asp103Argfs*25)	-	EC (1 †)
	c.2076_2077del, p.(Gly694Serfs*26)	*EOCRC* (*2: #23* and *#51*)	glioblastoma (1); OC (1)
	c.2305del, p.(Glu769Argfs*3)	-	Cancer-free (1)
	c.3106_3107del, p.(Lys1036Glyfs*7)	-	BC (1)
	**c.3406C** **>** T, p.(Arg1136*)	**EOCRC (1, additional, “EOCRC#89new”, diagnosed at 31 Y, DIGE-)**	-
*RECQL4* *NM_004260.3*	c.1048_1049del, p.(Arg350Glyfs*21)	-	BC (1)
	c.1573del, p.(Cys525Alafs*33)	*EOCRC* (*2: #57* and *#16*)	polyposis (1); cancer-free (1); BC (3)
	c.2269C>T, p.(Gln757*)	-	polyposis (1); BC (1); OC (1)
	c.2547_2548del, p.(Phe850Profs*33)	**EOCRC (1, additional, “EOCRC#88new”, diagnosed at 39 Y, DIGE-)**;	MBC (1)
	c.2590C>T, p.(Gln864*)	-	PC (1 ‡)
	c.2755+1G>A	-	BC (1)
	c.2994G>A, p.(Trp998*)	LOCRC (1, additional, diagnosed at 77 Y, DIGE-)	-
*RAD50* *NM_005732.3*	c.3229C>T, p.(Arg1077*)	-	BC (1)
	c.541dup, p.(Ser181Phefs*7)	-	BC (1)
	c.713_714insT, p.(Lys238Asnfs*8)	-	BC (1); OC (1); BC and OC (1); polyposis (1)
	c.2165dup, p.(Glu723Glyfs*5)	-	BC (1)
	c.3G>A, p.?	-	BC (1)
	c.2938_2942del, p.(Leu980*)	-	NA (1)
	c.1111dup, p.(Ile371Asnfs*14)	LOCRC (1, additional, diagnosed at 66 Y, †)	-
	c.354del, p.(Thr119Leufs*11)	-	BC (1)
	c.3489_3495del, p.(Glu1164Glyfs*22)	-	BC (1)
	c.2034del, p.(Gln678Hisfs*42)	LOCRC (1, additional, diagnosed at 68 Y, with polyposis, DIGE-)	BC (1); cancer-free (1)
	c.130-1G>T	*EOCRC* (*1: #59*)	BC (1)
	c.2801dup, p.(Asn934Lysfs*10)	-	BC (1)
	c.1281dup, p.(Gln428Thrfs*4)	-	BC (1)
	c.1704del, p.(Tyr569Ilefs*29)	-	BC and meningioma (1)
	c.2985_2989del, p.(Glu995Aspfs*3)	-	BC (1)
*RAD51C* *NM_058216.2*	c.1026+5_1026+7del	-	OC (2)
	c.31del, p.(Gln11Serfs*5)	-	BC (1)
	c.709C>T, p.(Arg237*)	-	BC (1); OC (1)
	c.965+5G>A	-	BC (2)
	c.773G>A, p.(Arg258His)	*EOCRC* (*1: #20*)	*-*
	c.414G>C, p.(Leu138Phe)	LOCRC (1, additional, diagnosed at 50 Y, DIGE-)	BC (2); MBC (1); cancer-free (1);
	c.577C>T, p.(Arg193*)	-	EC (1)
	c.358dup, p.(Thr120Asnfs*35)	-	OC (1); cancer-free (1)
	c.705+1G>A	-	OC (2)
	c.732del, p.(Ile244Metfs*9)	-	OC (1); melanoma (1)
	c.904+5G>T	-	BC (1)
	c.656dup, p.(Leu219Phefs*33)	-	OC (1)
	c.837+2T>C	-	BC (2)
	c.837+1G>T	-	BC (1)
	c.878del, p.(Asn293Ilefs*9)	-	Gastric cancer (1)
*ETV1* *NM_004956.4*	c.1378dup, p.(Met460Asnfs*80)	*EOCRC* (*1: #55*)	MBC (1); PC (1)
*HSD3B2* *NM_000198.3*	c.776C>T, p.(Thr259Met)	*EOCRC* (*1: #44*)	*-*
*LTBP2* *NM_000428.2*	c.4933C>T, p.(Arg1645Trp)	*EOCRC* (*1: #21*)	*-*
	c.468_469insG, p.(Thr157Aspfs*69)	-	BC (1)
	c.4721-1G>C	-	BC (1)
	c.709C>T, p.(Arg237*)	-	BC (1)
	c.2789-1G>A	-	OC (1)
*USP6* *NM_001304284.1*	c.3228+1G>A	*EOCRC* (*1: #44*)	BC (2)
	c.-1-2A>G	CRC (1, diagnosed at 47 Y, †)	BC (2); OC (1);
	c.3752dup, p.(Ser1252Glnfs*12)	-	BC (2); prostate cancer (1)
	c.349C>T, p.(Gln117*)	-	BC (2); OC (1)
	c.2828G>A, p.(Arg943His)	-	BC (1)
	c.2828G>T, p.(Arg943Leu)	-	BC (1)
	c.1337G>A, p.(Trp446*)	-	Cancer-free (1)
	c.79C>T, p.(Arg27*)	-	BC (1)
	c.4119C>G, p.(Tyr1373*)	-	BC (1); melanoma (1)
	c.3028C>T, p.(Gln1010*)	CRC (1, diagnosed at 47 Y, DIGE-)	BC (1)
	c.2866C>T, p.(Arg956*)	-	BC (1)
*EPHA10* *NM_001099439.1*	c.2446G>T, p.(Glu816*)	*EOCRC* (*1: #03*)	BC (3); OC (1); cancer-free (1)
	c.2920del, p.(Val974*)	-	OC (2)
	c.1066C>T, p.(Arg356*)	-	BC (2)
	c.851-1G>C	-	BC (1)
*NCOA1* *NM_003743.4*	c.4282dup, p.(Gln1428Profs*14)	*EOCRC* (*1: #05*)	*-*
	c.3304-2A>G	-	BC (1)

Germline variants were filtered and classified as described in the “Materials and Methods” section. This screen identified 2 additional EOCRCs (shown in bold): one with a heterozygous *NUTM1* non-sense variant and the other with a heterozygous *RECQL4* frameshift variant. These 2 “new” EOCRC patients (DIGE-) were included in the NGS database after the arbitrary 2021 cut-off date specified for the EOCRC cohort. LOCRC patients in this table were referred to the IUCT-O after 2021. BC: female breast cancer; CRC: colorectal cancer; EC: endometrial cancer; EOCRC: early-onset colorectal cancer (≤40 Y); HV: high-impact variant, i.e., truncating, splice or pathogenic/likely pathogenic variants; LOCRC: late-onset colorectal cancer (≥50 Y); MBC: male breast cancer; NA: phenotype not available; OC: ovarian cancer; PC: pancreas cancer; Y: years of age; †: carrier of Lynch syndrome due to a pathogenic variant of *MSH6*, *MSH2* or *PMS2;* ‡: *PALB2* pathogenic variant carrier.

## Data Availability

Patient germline genetic data generated in the context of healthcare are stored in the Oncogenetics Laboratory of the IUCT-Oncopole in Toulouse, and are not openly accessible, in accordance with French law (Civil Code and Bioethics Act). However, we can provide information on reasonable request. Data relevant to the present work are presented in the main text, and additional information (in particular on VUSs in the EOCRC and LOCRC cohorts) is provided as Supporting Information.

## Supplementary Materials

The following supporting information can be downloaded at: https://www.mdpi.com/article/10.3390/ijms26104672/s1.

## Abbreviations

The following abbreviations are used in this manuscript:

ADK	Adenocarcinoma
ALL	Acute lymphocytic leukemia
BGS	Baller–Gerold syndrome
CADD	Combined Annotation Dependent Depletion score
CH	Clonal hematopoiesis
CLL	Chronic lymphocytic leukemia
CNV	Copy number variant
CRC	Colorectal cancer
dMMR	Mismatch repair deficient
EOCRC	Early-onset colorectal cancer
HVs	High-impact variants: TVs, SVs or variants known to be PVs/LPVs
IHC	Immunohistochemistry
LPV	Likely pathogenic variant
LOCRC	Late-onset colorectal cancer
LNV	Likely neutral variant
MAF	Minor allele frequency
MSI-H	High microsatellite instability
MSI-L	Low microsatellite instability
MSS	Microsatellite stable
NET	Neuroendocrine tumor
NFE	Non-Finnish European
NGS	Next Generation Sequencing
NV	Neutral variant
pMMR	Mismatch repair proficient
PV	Pathogenic variant
RTS	Rothmund–Thomson syndrome
SV	Splice variant
TVs	Truncating variants, i.e., frameshift and non-sense variant
VAF	Variant allele frequency
VUS	Variant of unknown significance
WES	Whole exome sequencing
